# Supplementary material for: A Detailed Catalogue of Multi-Omics Methodologies for Identification of Putative Biomarkers and Causal Molecular Networks in Translational Cancer Research
Source: Int J Mol Sci. 2021 Mar 10;22(6):2822. doi: 10.3390/ijms22062822 (PMC8000236; doi:10.3390/ijms22062822)
Supplement: Supplementary file 1 [file ijms-22-02822-s001.zip › Supplementary_revised/Supplement_revised.docx]

Supplement

Supplementary Methods: Literature mining workflow

This document provides the code of the systematic literature search performed as part of the review: Evaluation of multi-omics methodologies and tools for identification of putative biomarkers and causal molecular networks in translational cancer research. The systematic Literature research is based on applying Entrez Direct searches in the PubMed database. See the documentation of this tool: <https://www.ncbi.nlm.nih.gov/books/NBK179288/>.

The following sections describe the executed commands and filtering strategy. Please note that the integration of multi-omics data is a growing field and the output by numbers will look different in numbers in the future as publications are added continuously. This search has been performed on the 10th of November 2020.

## General literature search

This systematic approach starts with a more general search, which enables downstream filtering. We evaluated the available literature considering the various different phrasings and synonyms used in publication title and abstract. Importantly, we observed variable spelling of multi-omics, such as multi omics or multiomics, which we took into account. This search resulted in 755 papers. This dataset is the base for further filtering and detailed classification into one of the five classes.

*# This run gives you a summary of literature in English and provides the Pubmed ID, title, and publication date*
esearch -db pubmed -query "((multi AND omics) OR multi-omics OR multiomics OR (multivariate AND genomic) OR (Algorithms AND integrative AND Cluster AND Analysis)) AND data AND integration" **|** efilter -query "english [LANG]" **|** efetch -format docsum **|** xtract -pattern DocumentSummary -element Id Title PubDate

## Filtering for supervised methods

The general dataset should contain the majority of multi-omics related papers. For a more methodology-related filtering, we tried to distinguish between supervised and unsupervised methods. Therefore, we added additional filter criteria for supervised methods in the efilter section of the command below. We added the word supervised into this section as it searches for this term in abstracts or titles of publications.

*# This run gives you a filtered list for supervised approaches in literature in English and provides Pubmed IDs*
esearch -db pubmed -query "((multi AND omics) OR multi-omics OR multiomics OR (multivariate AND genomic) OR (Algorithms AND integrative AND Cluster AND Analysis)) AND data AND integration" **|** efilter -query "english [LANG] AND supervised" **|** efetch

## Filtering for unsupervised methods

In order to filter for unsupervised multi-omics data integration related papers, we added the words unsupervised, cluster, factor analysis and excluded the term supervised in the efilter option of the command below.

*# This run gives you a filtered list for unsupervised approaches in literature in english and provides Pubmed IDs*
esearch -db pubmed -query "((multi AND omics) OR multi-1omics OR multiomics OR (multivariate AND genomic) OR (Algorithms AND integrative AND Cluster AND Analysis)) AND data AND integration" **|** efilter -query " english [LANG] AND (unsupervised OR cluster OR (Factor AND Analysis) NOT supervised)" **|** efetch

## Filtering for reviews

In addition, we searched for multi-omics data integration related reviews in order to get an overview of review articles in the field of interest. We searched for the publication type review and the word review as it may occur in abstracts or titles of publications.

*# This run gives you a filtered list for reviews in literature in english and provides Pubmed IDs*
esearch -db pubmed -query "((multi AND omics) OR multi-omics OR multiomics OR (multivariate AND genomic) OR (Algorithms AND integrative AND Cluster AND Analysis)) AND data AND integration" **|** efilter -query "english [LANG] AND (review [PTYP] OR review)" **|** efetch

## Filtering for tools

In order to get an idea of how many papers are prior dealing with tools for multi-omics data integration, we added the words tool, application, algorithm, and method to the filtering criteria.

*# This run gives you a filtered list for tools in literature in english and provides Pubmed IDs*
esearch -db pubmed -query "((multi AND omics) OR multi-omics OR multiomics OR (multivariate AND genomic) OR (Algorithms AND integrative AND Cluster AND Analysis)) AND data AND integration" **|** efilter -query "english [LANG] AND (Tool OR Application OR Algorithm OR method)" **|** efetch

## Filtering for cancer context

As our application focus is cancer we wanted to know how many of the mined papers are related to cancer research, so we added human and cancer as a MESH terms and also cancer as a search term here.

*# This run gives you a filtered list concerning cancer in literature in English and provides Pubmed IDs*
esearch -db pubmed -query "((multi AND omics) OR multi-omics OR multiomics OR (multivariate AND genomic) OR (Algorithms AND integrative AND Cluster AND Analysis)) AND data AND integration" **|** efilter -query " english [LANG] AND ((humans [MESH] AND cancer [MESH]) OR cancer)" **|** efetch

Supplementary Figures


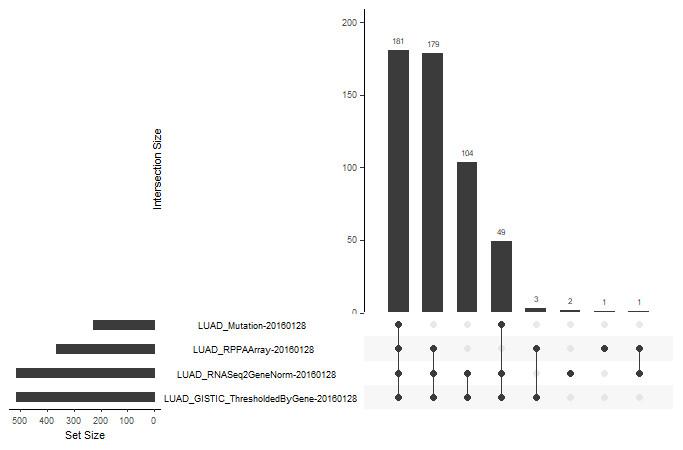
Supplementary Figure S1: Upset plot on LUAD data availability, showing how many LUAD samples have data for each combination of retrieved omics layers. In this dataset, 181 samples have all four assays, 179 are missing mutations, 104 have only gene expression (RNASeq2GeneNorm) and CNVs (GISTIC), etc. The plot was created with the R package UpsetR.

Supplementary Figure S2: Classification of Literature from systematic literature research. The literature research included five classification schemes (Unsupervised, Supervised, Cancer, Review, and Tools), each with specific filtering criteria. An additional category in this graph displays the occurrence of any of these classifications.


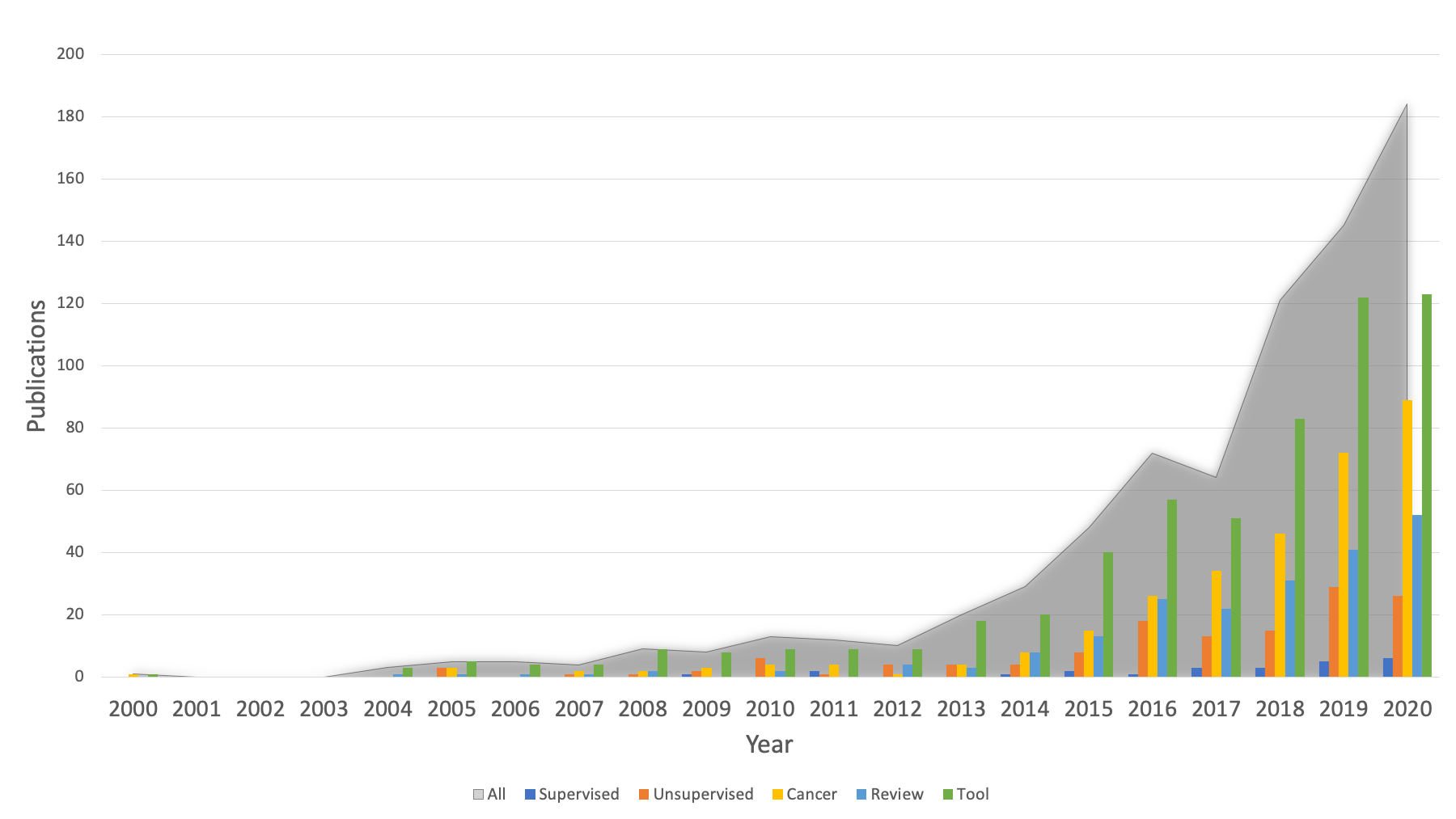


Supplementary Figure S3: Distribution by year of publications dealing with multi-omics data integration and subcategories from the literature mining approach.


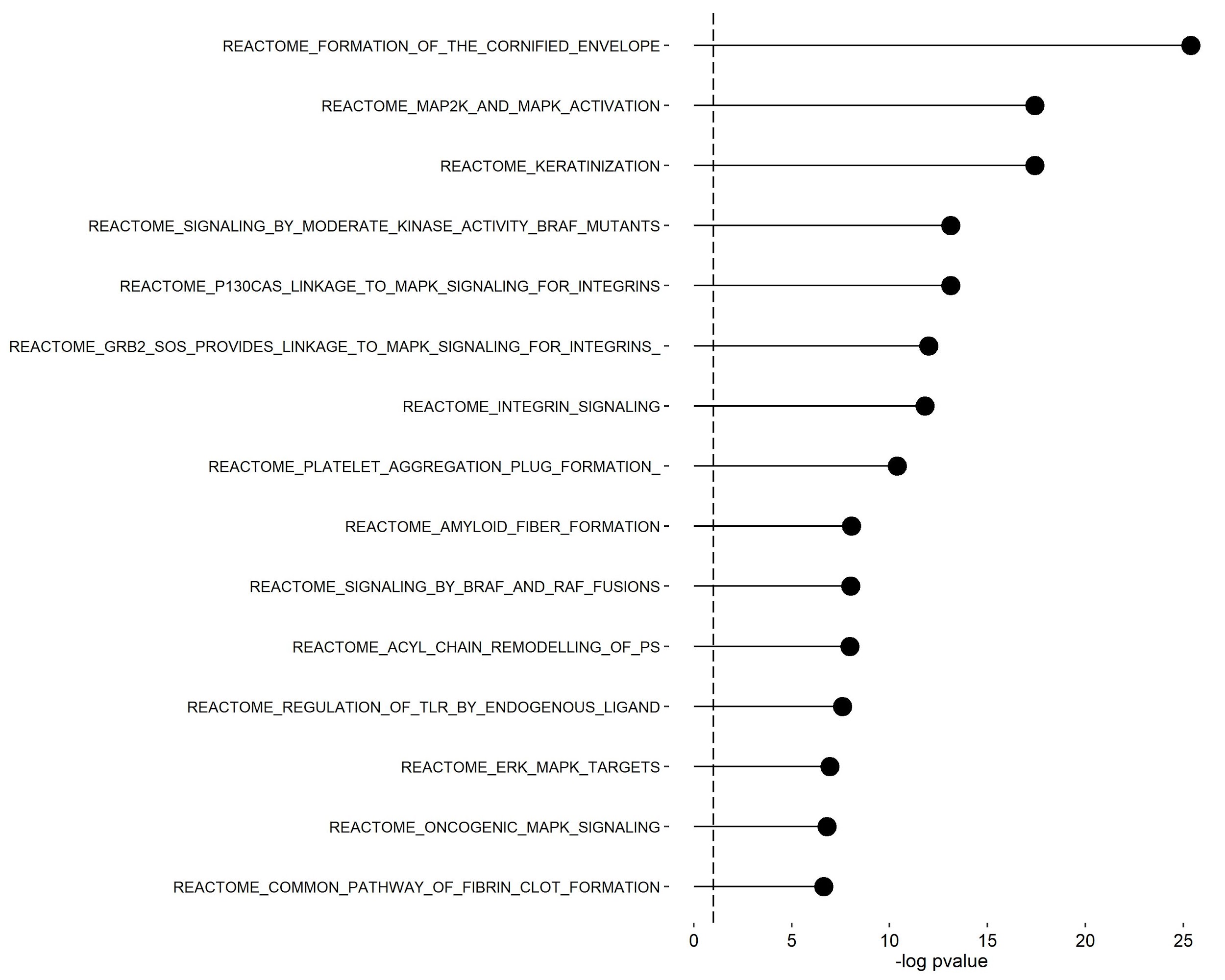


Supplementary Figure S4: Visualization of Factor 3 functional enrichment analysis results, from running GSEA on MOFA factor 3 with gene expression negative weights and Reactome gene sets. In this plot the top 15 ranked Reactome pathways are shown, based on the resulting p-values depicted on the x-axis. The overall majority of the pathways are associated with MAPK signalling cascades.


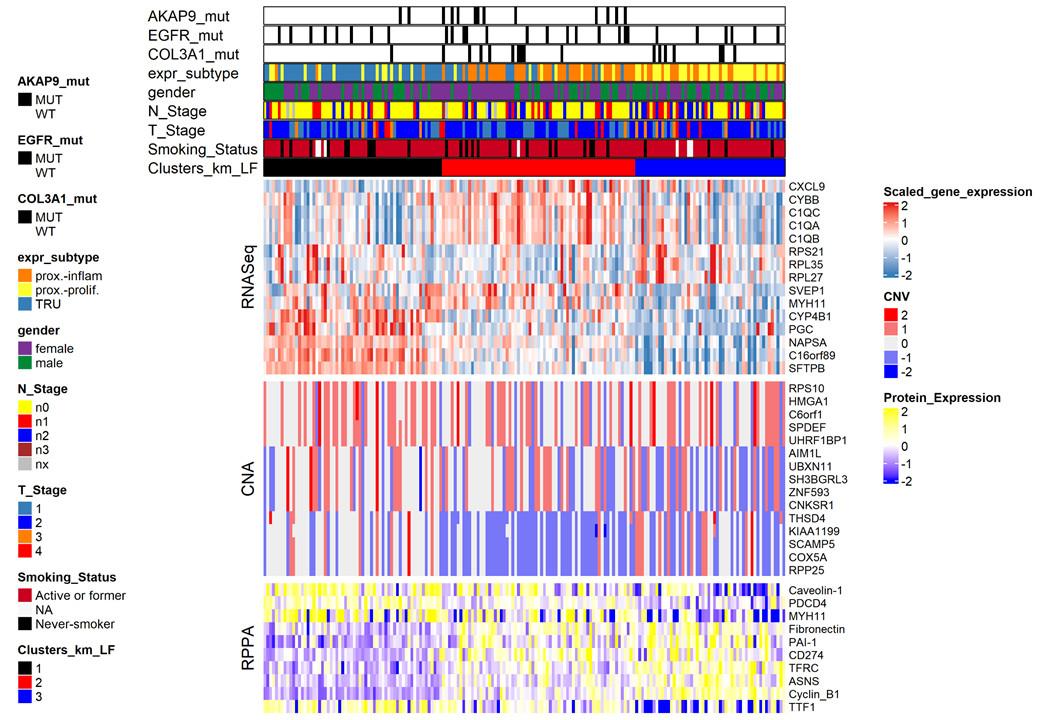


Supplementary Figure S5: Comparative heatmap plots of selected multi-omics features, based on the MOFA analysis results (181 cancer samples, ComplexHeatmap R package). Samples have been separated into 3 distinct groups based on the “multi-omics” clustering approach. For each of the three omics layers, the most “significant” features are shown, based on the relative feature weights from each MOFA factor. Features have been re-ordered by the method of hierarchical clustering (“complete” method, “euclidean” distance). For the gene expression heatmap samples with relatively high expression of a given gene are marked in red and samples with relatively low expression are marked in blue. Similarly, for the distinct values of the copy number variation data red color denotes amplification, whereas the blue deletion. Finally, for the RPPA (protein) data, blue color represents under-abundance and yellow over-abundance, respectively.

*
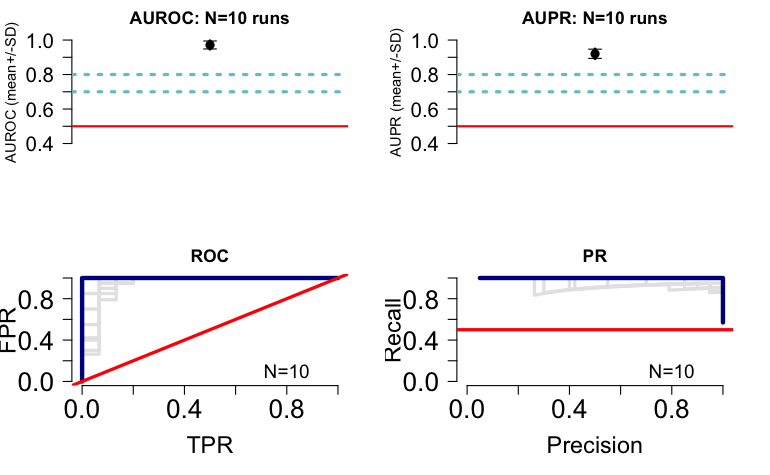
*

Supplementary Figure S6: Evaluation of netDx performance on the CLL cohort. Upper graphs show the mean area across 10 train/test splits and the standard deviation of performed binary classification (left AUROC (Area Under the Receiver Operating Characteristics) and right PR (Precision-Recall)). Plots at the bottom are showing the AUROC plot (left; FPR = False Positive Rate, TPR = True Positive Rate) and the PR plot (right).


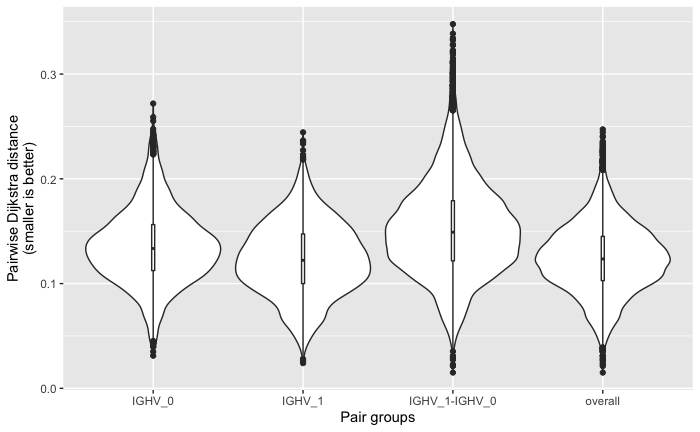


Supplementary Figure S7: Illustration of pairwise Dijkstra distance within and among IGHV patient classes of the integrated patient similarity network (IGHV_0 = no IGHV mutation, IGHV_1 = IGHV mutation).

Supplementary Files

Supplementary Table S1: Summary of results from the performed literature search including Pubmed ID, date, title, and classification into supervised, unsupervised, cancer, review or tool.
